# Supplementary material for: A Vegetarian Diet Significantly Changes Plasma Kynurenine Concentrations
Source: Biomolecules. 2023 Feb 18;13(2):391. doi: 10.3390/biom13020391 (PMC9953657; doi:10.3390/biom13020391)
Supplement: Supplementary file 1 [file biomolecules-13-00391-s001.zip › biomolecules-2221540-supplementary.pdf]

Assessment of disease risk
by targeted metabolomics

Supplementary data on method for analysis of Picolinic acid (Pic)

Method based on article
Midttun et al (2009), PMID 19337982.

Material
Picolinic acid (purity >98%) was obtained from Sigma-Aldrich, St.Louis, MO 63178 USA.
2 Picolinic-d4 acid (purity >98%) was obtained from CDN isotopes. Ponte-Claire, Quebec, Canada.

Instrumentation
Same as in PMID 19337982.

Chromatography and detection
LC-MS/MS; positive-ion multiple reaction monitoring (MRM); retention time = 2.25 min.
Picolinic acid precursor ion = 124 m/z; product ion = 78 m/z.
Picolinic-d4 acid precursor ion = 128 m/z; product ion = 82 m/z.

Method performance
Linear range: 8 - 400 nmol/L.
Linearity: r2: 0.99.
LOD (S/N >5): 8 nmol/L.
Within-day CV: 6-7 %.
Between-day CV: 5-8 %.
